# Aggrecan Hypomorphism Compromises Articular Cartilage Biomechanical Properties and Is Associated with Increased Incidence of Spontaneous Osteoarthritis

**DOI:** 10.3390/ijms20051008

**Published:** 2019-02-26

**Authors:** Paolo Alberton, Hans Christian Dugonitsch, Bastian Hartmann, Ping Li, Zsuzsanna Farkas, Maximilian Michael Saller, Hauke Clausen-Schaumann, Attila Aszodi

**Affiliations:** 1 Laboratory of Experimental Surgery and Regenerative Medicine, Clinic for General, Trauma and Reconstructive Surgery, Ludwig-Maximilians University, 80336 Munich, Germany; Hans.Dugonitsch@med.uni-muenchen.de (H.C.D.); bastian.hartmann@hm.edu (B.H.); Ping.Li@med.uni-muenchen.de (P.L.); Zsuzsanna.Farkas@med.uni-muenchen.de (Z.F.); Maximilian.Saller@med.uni-muenchen.de (M.M.S.); Attila.Aszodi@med.uni-muenchen.de (A.A.); 2 Center for Applied Tissue Engineering and Regenerative Medicine, Munich University of Applied Sciences, 80533 Munich, Germany; hauke.clausen-schaumann@hm.edu;; 3 Center for NanoScience, Ludwig-Maximilians University Munich, 80799 Munich, Germany

**Keywords:** aggrecan, articular cartilage, osteoarthritis, atomic force microscopy

## Abstract

The gene encoding the proteoglycan aggrecan (*Agc1*) is abundantly expressed in cartilage during development and adulthood, and the loss or diminished deposition of the protein results in a wide range of skeletal malformations. Furthermore, aggrecan degradation is a hallmark of cartilage degeneration occurring in osteoarthritis. In the present study, we investigated the consequences of a partial loss of aggrecan in the postnatal skeleton and in the articular cartilage of adult mice. We took advantage of the previously described *Agc1^tm(IRES-CreERT2)^* mouse line, which allows for conditional and timely-regulated deletion of floxed, cartilage-expressed genes. As previously reported, the introduction of the CreER^T2^ cassette in the 3’UTR causes a disruption of the normal expression of *Agc1* resulting in a hypomorphic deposition of the protein. In homozygous mice, we observed a dwarf phenotype, which persisted throughout adulthood supporting the evidence that reduced aggrecan amount impairs skeletal growth. Homozygous mice exhibited reduced proteoglycan staining of the articular cartilage at 6 and 12 months of age, increased stiffening of the extracellular matrix at six months, and developed severe cartilage erosion by 12 months. The osteoarthritis in the hypomorph mice was not accompanied by increased expression of catabolic enzymes and matrix degradation neoepitopes. These findings suggest that the degeneration found in homozygous mice is likely due to the compromised mechanical properties of the cartilage tissue upon aggrecan reduction.

## 1. Introduction

Osteoarthritis (OA) is a degenerative disorder of the synovial joints causing significant disability and morbidity in the aging population. OA is the most common cause of disability in elderly with an estimated prevalence of 10–15% of adults aged over 60. According to the United Nations, 130 million people will suffer from OA by 2050, and among them one-third being severely disabled. Genetic and numerous environmental risk factors (e.g., obesity, trauma, joint overuse) are responsible for the etiology of OA. Despite a widespread awareness of the disease, the pathogenesis of OA is not completely understood. It is generally accepted that uncontrolled homeostasis of the extracellular matrix (ECM) produced by chondrocytes is critical for the onset of the condition. Changes in the composition and structural organization of cartilage ECM proteins may activate catabolic processes degrading proteoglycans and the heterotypic collagen II/IX/XI fibrils, which in turn alter the biomechanical properties of the tissue compromising the correct function of articular cartilage (AC), therefore laying the foundations for the establishment of the disorder.

The large aggregating protein aggrecan is the most abundant proteoglycan of the typical cartilaginous structures such us the growth plate and the articular cartilage (reviewed in [1,2]). The 250 kDa aggrecan protein core is composed of three globular (G1, G2 and G3), and two interglobular domains. The large interglobular region between the G2 and G3 domains carries about 100 chondroitin sulphate (CS) and 30 keratan sulphate (KS) chains [3]. The high negative charge density of the CS chains attracts counter ions and draw water molecules into the tissue, thus creating a positive osmotic pressure which endows articular cartilage to resist compressive forces. Aggrecan molecules form huge tertiary complexes in the cartilage ECM through interaction of the N-terminal G1 domain with hyaluronan and link protein. The KS chains interact with fibrillar collagens [4], and the C-terminal G3 domain binds to multiple ECM proteins including tenascins and fibulins [1]. Apart from creating the basis for the viscoelastic properties of cartilage, aggrecan–hyaluronan aggregates with their numerous glycosaminoglycan chains are playing important roles in cell-ECM crosstalk, binding and release of growth factors and morphogens [5,6]. In normal cartilage, aggrecan gene expression is sustained and its protein turnover relatively rapid [7,8].

The critical importance of aggrecan for endochondral bone formation and the function of permanent cartilage structures is demonstrated by numerous mutations identified in the gene coding for aggrecan in both human and various animal species (reviewed in [9]). Mutations in the human aggrecan gene (*ACAN or AGC1*) lead to a broad range of non-lethal skeletal dysplasia including spondyloepimetaphyseal dysplasia (SEMD) [10], osteochondritis dissecans with early onset of OA [11], and various short stature syndromes with accelerated bone maturation [12]. It has been recently suggested that human aggrecanopathies are mechanistically caused by *AGC1* mutations resulting either in haploinsufficiency or disruption of the cartilage structure [9]. In animals including mice, chicken and cattle, naturally occurring homozygous null or functional null mutations result in embryonic lethality, while heterozygous animals display milder skeletal dysplasias [1,9]. In mice, the *cmd* (*cartilage matrix deficiency*) and the *cmd–bc* deletion mutations lead to truncated aggrecan molecules or the lack of aggrecan, respectively, and the lethal phenotype is characterized by disproportionate dwarfism, short snout, protruding tongue and enlarged abdomen [13,14]. Heterozygous *cmd/+* mice are hypomorphic showing a reduced aggrecan mRNA level in embryonic limb cartilage (81% of the wild type), and develop postnatal dwarfism, spinal misalignment and age-associated intervertebral disc degeneration [15].

In modern functional genetics, generation of mouse lines which allow inducible gene deletion is of great importance for studying gene function and generating animal models for human disorders. Recently, the *Agc1^tm(IRES-CreERT2)^* mouse line, further referred to here as *Agc1^CreERT2^*, has been established for inducible, conditional inactivation of genes floxed with *loxP* elements in chondrocytes [16]. In this model, a transgene encoding the fusion polypeptide of the Cre recombinase and a mutant ligand binding domain of the estrogen receptor (*CreER^T2^*) has been introduced (knocked in) into the 3’ untranslated region (UTR) of the endogenous mouse *aggrecan* gene (*Agc1*). These mice represent a precious tool, allowing the inducible inactivation of genes involved in cartilage degenerative disorders in a spatially and temporally coordinated manner after tamoxifen administration [17,18,19,20,21,22,23,24,25,26]. However, we have noticed during breeding of the line in our animal facility that the homozygous knock-in mice develop a postnatal dwarf phenotype. Indeed, it has been recently published that *Agc1^CreERT2/CreERT2^* mice have reduced body weight and length, and shorter endochondral bones compared to wild type and heterozygous mice [27]. Histological analysis indicated reduced height of the growth plate and the articular cartilage. Further molecular and biochemical investigation revealed that the homozygous animals express about 50% of both aggrecan mRNA and protein in cartilage compared to the normal levels in wild type [27]. It was suggested that the decreased *Agc1* expression may have been caused by the deletion of a 760 bp region of the 3’UTR, disrupting putative regulatory regions or by the negative effect of the targeting construct-derived *pgk* promoter on the activity of the endogenous *Agc1* promoter [27]. As a consequence of the disturbed skeletal growth in *Agc1^CreERT2/CreERT2^* mice, only heterozygous *Agc1^CreERT2/+^* mice were suggested to be used for conditional deletion of the floxed genes in chondrocytes [27].

In this study, we extended the analysis of the *Agc1^CreERT2^* mice for the function of the articular cartilage. We found that *Agc1^CreERT2/CreERT2^* mice develop severe cartilage erosion of the knee joint at 12 months of age. Importantly, heterozygous *Agc1^CreERT2/+^* mice also exhibited a tendency for increased articular cartilage destruction compared with wild type animals. Applying nanoscale indentation-type atomic force microscopy (IT-AFM) to characterize cartilage biomechanics, we demonstrated that *Agc1^CreERT2/CreERT2^* mice have stiffer cartilaginous ECM in the superficial, middle and deep zones of the AC compared to control and heterozygous mice. We believe that this alteration in biomechanical properties compromises the basic cushioning function of AC, eventually predisposing mice to develop spontaneous OA. Thus, the *Agc1^CreERT2/CreERT2^* mouse is a valuable model to investigate articular cartilage degradation in dependence of aggrecan level.

## 2. Results

### 2.1. Agc1^CreERT2/CreERT2^ Dwarf Phenotype Persist through the Adulthood

**Figure 1:**
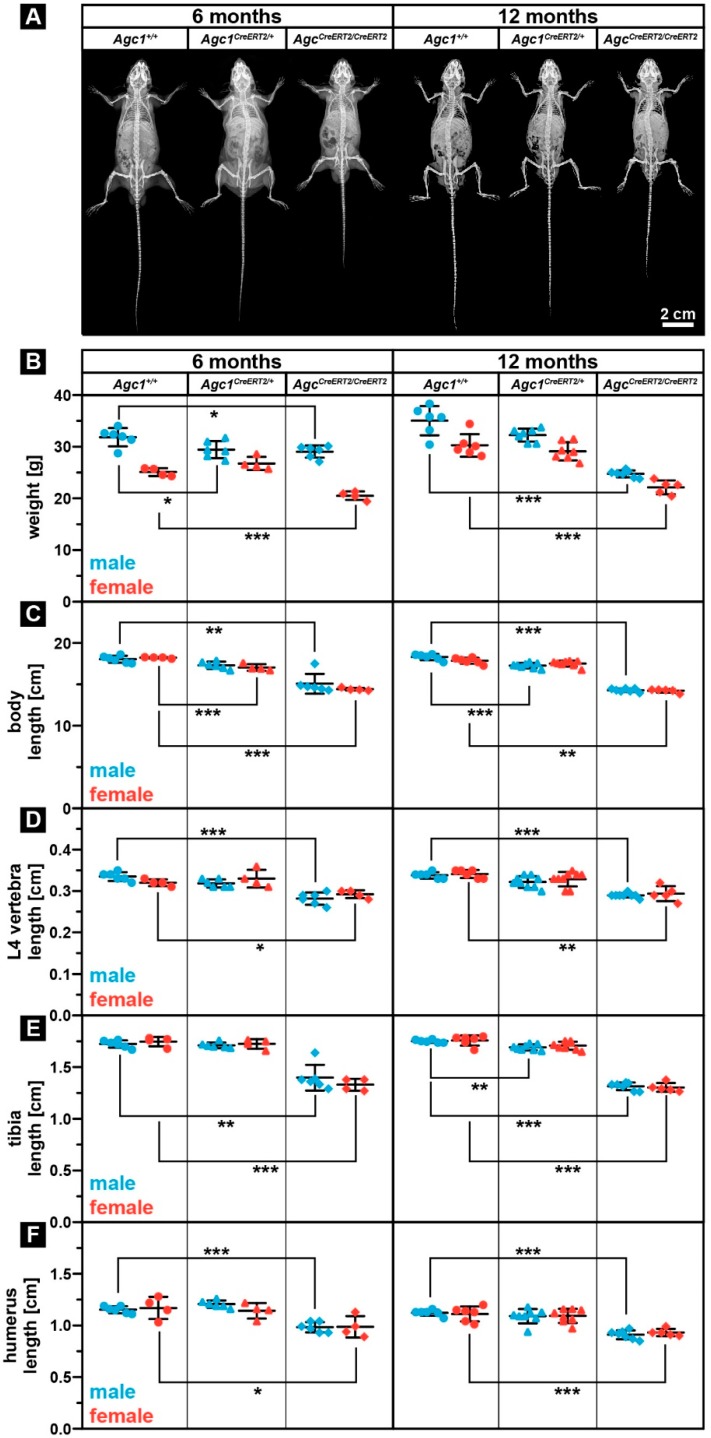
*Agc1^CreERT2/CreERT2^* mice develop dwarfism which is maintained after skeletal maturation. (**A**) representative X-ray images of *Agc1^+/+^*, *Agc1^CreERT2/+^* and *Agc1^CreERT2/CreERT2^* mice at 6 and 12 months of age. (**B**–**F**) analysis of body weight (**B**), body length (**C**), L4 vertebra length (**D**), tibia length (**E**) and humerus length (**F**) at 6 and 12 months of age. Bars represent the mean ± standard deviation (SD). *n* > *Agc1^+/+^*: 6(m)/4(f); *Agc1^Cre/+^*: 6(m)/4(f); *Agc1^Cre/Cre^*: 6(m)/4(f) at six months; and *n* > *Agc1^+/+^*: 6(m)/6(f); *Agc1^Cre/+^*: 7(m)/7(f); *Agc1^Cre/Cre^*: 7(m)/5(f) at 12 months. Statistical significance calculated by one-way ANOVA where * *p* < 0.5; ** *p* < 0.01; *** *p* < 0.001.

In our breeding colony, we have observed that, after two weeks of age, both male and female *Agc1^CreERT2/CreERT2^* mice were consistently smaller compared to wild type confirming the previously reported early postnatal dwarfism between one and three months [27]. We did not notice any other gross visual abnormalities up to one year of age: the *Agc1^CreERT2/CreERT2^* mice had apparently normal gait and physical activity. X-ray analysis confirmed the dwarf phenotype of *Agc1^CreERT2/CreERT2^* mice at 6 and 12 months of age without an apparent sign of spinal misalignment (Figure 1A).

In order to assess body parameters after the skeletal maturation, we have analyzed body weight, body length, the length of the fourth lumbar (L4) vertebra, and the length of long bones of the appendicular skeleton (tibia, femur and humerus) of homozygous *Agc1^CreERT2/CreERT2^*, heterozygous *Agc1^CreERT2/+^* and wild type *Agc1^+/+^* animals at ages of 6 and 12 months. The body weight of both genders was significantly lower in *Agc1^CreERT2/CreERT2^* mice compared to *Agc1^+/+^* mice (males: 9% and 29% reduction at 6 and 12 months, respectively; females: 18% and 27% reduction at 6 and 12 months, respectively) (Figure 1B). Heterozygous *Agc1^CreERT2/+^* mice showed the tendency for reduced weight, which, however, only reached significance in 6-month-old males (7.6% reduction compared to wild type). X-ray micrographs were used to measure the length of the body (from snout to the end of the tail), the L4 vertebra, tibia, femur, and humerus, as characteristics for the size of the skeleton (Figure 1C,F and not shown). The body length of both gender was significantly reduced in *Agc1^CreERT2/CreERT2^* mice compared to *Agc1^+/+^* mice (males: 17% and 22% reduction at 6 and 12 months, respectively; females: 21% and 20% reduction at 6 and 12 months, respectively) (Figure 1C). Similarly, we found a statistical significant decrease in the length of the L4 vertebra when comparing *Agc1^CreERT2/CreERT2^* homozygous to control mice of both sex (males: 16% and 14% reduction at 6 and 12 months, respectively; females: 9% and 14% reduction at 6 and 12 months, respectively) (Figure 1D). Regarding long bones, the length of the total tibia was significantly decreased in both male and female *Agc1^CreERT2/CreERT2^* mice comparing to *Agc1^+/+^* mice (males: 17% and 22% reduction at 6 and 12 months, respectively; females: 21% and 20% reduction at 6 and 12 months, respectively) (Figure 1E). A similar trend was also observed in the length of the femur (data not shown). In the forelimbs, the length of the humerus was significantly reduced again in both male and female homozygous *Agc1^CreERT2/CreERT2^* mice compared to controls (males: 15% and 19% reduction at 6 and 12 months, respectively; females: 16% reduction at both 6 and 12 months) (Figure 1F). Interestingly, we found that heterozygous *Agc1^CreERT2/+^* mice occasionally exhibited significantly reduced skeletal parameters compared to *Agc1^+/+^* animals: 6% decreased body length in females at six months; 5.7% decreased body length in males at 12 months; 3.4% reduced tibia length in 12-month-old males (Figure 1C,E). Taken together, our results indicate that the dwarf phenotype of the *Agc1^CreERT2/CreERT2^* mice is maintained after the end of skeletal maturation (about four months of age) and there is no compensatory catch up during skeletal growth. It is important to note that heterozygous *Agc1^CreERT2/+^* mice displayed the tendency for reduced body weight, body length and tibia length at almost all investigated postnatal stages.

### 2.2. Agc1^CreERT2/CreERT2^ Mice Are Characterized by Reduced Aggrecan Expression

**Figure 2:**
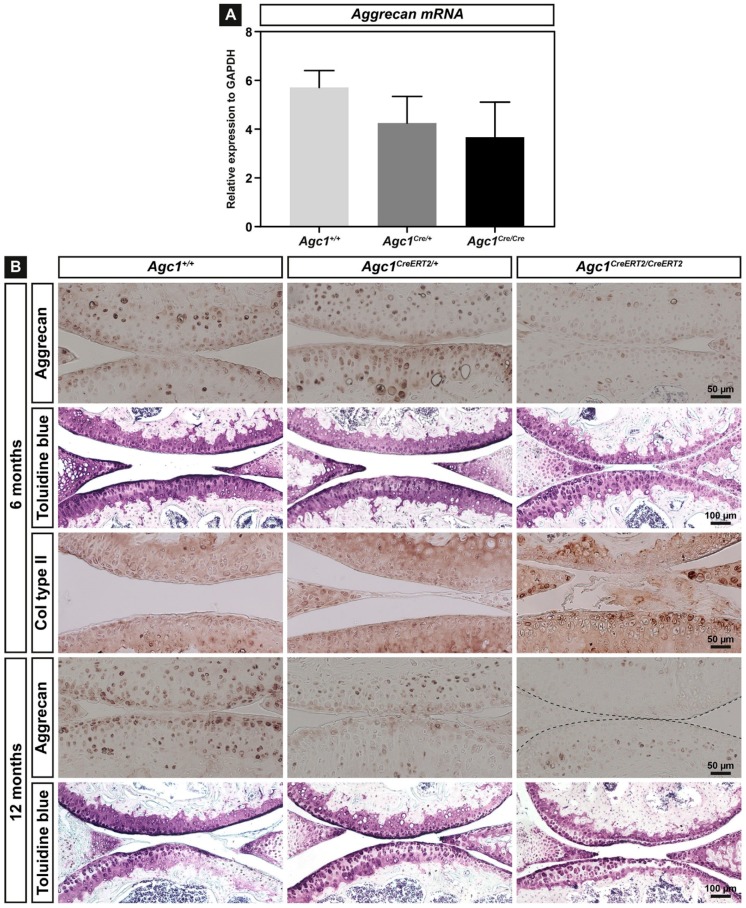
Expression of aggrecan in cartilage of mice carrying the *CreER^T2^* transgene. (**A**) relative gene expression of *Agc1* in newborn rib cartilage primary chondrocytes of *Agc1^+/+^*, *Agc1^CreERT2/+^* and *Agc1^CreERT2/CreERT2^*, mice. The mean values ± SD from three independent animals of each genotype are shown; (**B**) sagittal sections of the knee joint were stained with antibodies against aggrecan, collagen type II, and with Toluidine blue. *n* = 5 mice per genotype.

As the expression level of aggrecan could crucially influence the phenotype, we have analyzed aggrecan mRNA and protein by quantitative RT-PCR and immunohistochemistry, respectively, in all genotypes. We have isolated total RNA from newborn rib cartilage (*n* = 3 for each genotype) and performed qPCR using *Agc1*-specific TaqMan probe. The mRNA expression was reduced to 74% and 64% of the control in *Agc1^CreERT2/+^* and *Agc1^CreERT2/CreERT2^* mice, respectively, indicating that insertion of the *CreER^T2^* transgene into the 3´UTR affect total *aggrecan* gene expression in both homozygous and heterozygous knock-in animals (Figure 2A).

Next, we performed immunohistochemical staining in knee joints of 6- and 12-month-old animals using antibody against mouse aggrecan core protein (Figure 2B). The articular cartilage of the femur and tibia showed strong, predominantly pericellular/territorial matrix localization of the aggrecan protein in *Agc1^+/+^* mice at 6 and 12 months of age. In *Agc1^CreERT2/CreERT2^* mice, aggrecan was greatly reduced at 6 and 12 months of age, whereas, in *Agc1^CreERT2/+^* mice, aggrecan immunoreactivity was comparable to wild type at six months and decreased at 12 months. As the level of aggrecan core protein in the tissue correlates with the number of CS and KS chains, we also stained the knee joint sections with Toluidine blue at pH = 2.5 to detect sulphated glycosaminoglycans (sGAGs) (Figure 2B). The staining was intense in *Agc1^+/+^* and *Agc1^CreERT2/+^* cartilage, while the signal was reduced in *Agc1^CreERT2/CreERT2^* cartilage. Expression of cartilage fibril component type II collagen in the articular cartilage was comparable in each genotype.

Taken together, our expression analyses confirmed that *Agc1^CreERT2/CreERT2^* mice are hypomorph, characterized by reduced *Agc1* RNA expression in primary chondrocytes isolated from newborn rib cartilage and greatly diminished aggrecan protein deposition in adult articular cartilage.

### 2.3. Aggrecan Depletion Results in Stiffening of the Articular Cartilage

**Figure 3:**
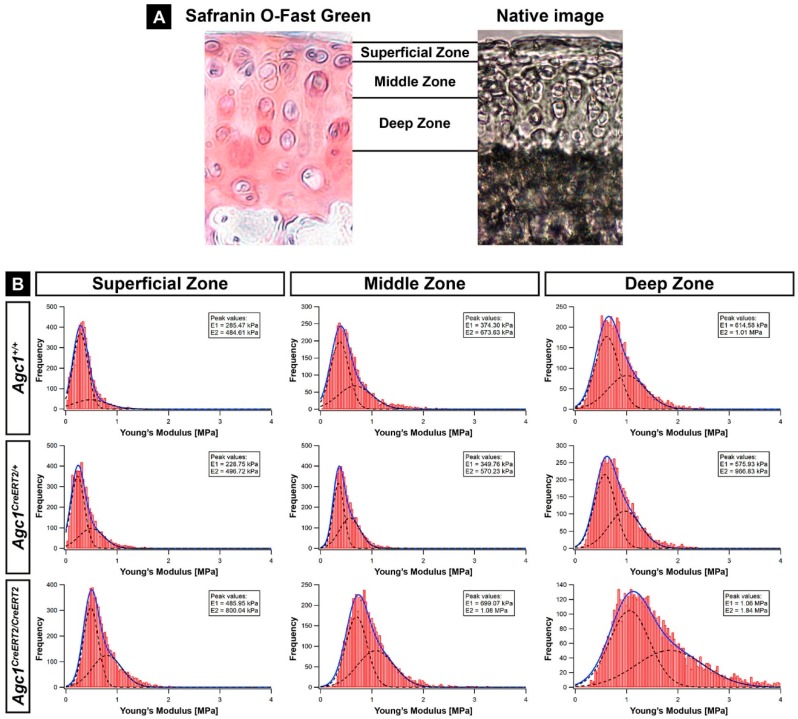
IT-AFM reveals stiffening of articular cartilage in *Agc1^CreERT2/CreERT2^* mice at six months of age. (**A**) Safranin Orange /Fast Green staining and an overview image of a native articular cartilage section show the zones assessed by IT-AFM (40× magnification); (**B**) histograms showing the stiffness distribution in various zones of the tibial plateau cartilage determined by nano-indentation (*n* = 3 animals in each group). On each histogram, the solid line represents the sum of two Gaussian functions, whereas the dashed lines indicate individual fits. The first and the second stiffness peaks (E1 and E2) represent the proteoglycan gel and the collagen fibrils, respectively. *n* = 3 animals per genotype.

Reduced deposition of aggrecan and sulphated GAGs in the cartilage of *Agc1^CreERT2/CreERT2^* mice may result in alteration of the biomechanical properties of the tissue. In order to assess this possibility, we applied nano-scaled indentation-type atomic force microscopy (IT-AFM) on native knee sections and recorded the compressive stiffness in the various zones (superficial, middle and deep) of the AC at six months of age (Figure 3A,B). For all measurements, we observed a bimodal distribution of the Young’s moduli, where the first peak (E1) on the histograms represents the softer proteoglycan moiety and the second peak (E2) is attributed to the stiffer collagen fibrils (Figure 3B) [28,29,30]. We found that, in the *Agc1^CreERT2/CreERT2^* mice, both peaks were shifted towards 60%–80% higher values in each AC zone compared to *Agc1^+/+^* mice indicating a general stiffening of the cartilage extracellular matrix. The values in wild type and *Agc1^CreERT2/CreERT2^* superficial zone were E1 = 285.47/E2 = 484.61 kPa vs. E1 = 485.95 kPa/E2 = 800.04 kPa; in the middle zone were E1 = 374.30 kPa/E2 = 673.63 kPa vs. E1 = 699.07 kPa/E_2_ = 1.08 MPa; in the deep zone were E1 = 614.58 kPa/E2 = 1.01 MPa vs. E1 = 1.06 MPa/E2 = 1.84 MPa. In contrast, *Agc1^CreERT2/+^* mice showed comparable stiffness values with *Agc1^+/+^* mice, in a range of 4% to 15% difference. All together, these data indicate that reduced amount of aggrecan in *Agc1^CreERT2/CreERT2^* mice severely compromises nanomechanical properties of the matrix, making the articular cartilage stiffer.

### 2.4. Agc1^CreERT2/CreERT2^ Mice Develop Spontaneous Osteoarthritis during Aging

**Figure 4:**
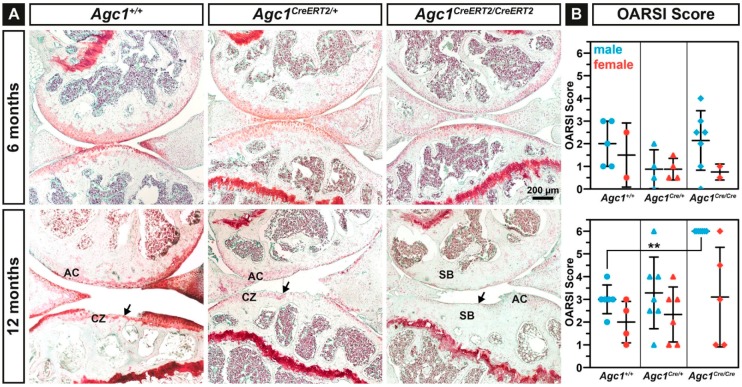
*Agc1^CreERT2/CreERT2^* mice exhibit severe cartilage erosion at 12 months of age. (**A**) representative images of Safranin O/Fast Green stained knee articular cartilage of 6 and 12 months old *Agc1^+/+^*, *Agc1^CreERT2/+^* and *Agc1^CreERT2/CreERT2^* mice. Arrows indicate severe degeneration of the articular cartilage. Abbreviations: AC, articular cartilage; CZ, calcified zone; SB, subchondral bone. (**B**) quantification of spontaneous cartilage degeneration using the OARSI scoring system. Bars represent mean ± SD. *n* = *Agc1^+/+^*: 5(m)/2(f); *Agc1^Cre/+^*: 4(m)/4(f); *Agc1^Cre/Cre^*: 7(m)/2(f) at six months; and *n* = *Agc1^+/+^*: 6(m)/4(f); *Agc1^Cre/+^*: 7(m)/6(f); *Agc1^Cre/Cre^*: 7(m)/5(f) at 12 months. Statistical significance calculated by one-way ANOVA, where ** *p* < 0.01.

The development of articular cartilage deterioration was examined in control, heterozygous and homozygous knock-in animals at 6 and 12 months of age (Figure 4A). Safranin O-Fast Green staining on serial knee joint sections revealed normal or mildly affected articular cartilage at six months in each genotypes. At 12 months, all male *Agc1^CreERT2/CreERT2^* mice (*n* = 7) showed complete degeneration of the articular cartilage with exposure of the subchondral bone (arrow). At this age, wild type and heterozygous *Agc1^CreERT2/+^* mice frequently exhibited clefts extending into the calcified zone (arrow).

Quantification of the cartilage damage according to the Osteoarthritis Research Society International (OARSI) histopathological recommendations (Figure 4B) revealed mildly decreased mean score in male *Agc1^CreERT2/+^* mice compared to control and homozygous knock-in mice at six months of age. At 12 months, male but not female *Agc1^CreERT2/CreERT2^* mice displayed significantly increased mean erosion score compared to wild type (males: 6.000 vs. 3.000, ** *p* < 0.01; females: 3.100 vs. 2.000). Control and heterozygous *Agc1^CreERT2/+^* mice showed similar mean cartilage erosion scores (male: 3.000 vs. 3.285; female: 2.000 vs. 2.333) in both genders. However, male heterozygous *Agc1^CreERT2/+^* mice had the tendency to develop more severe osteoarthritic damages exhibiting 25–50% erosion or complete eburnation of the AC surface.

In order to assess the possibility that the onset of cartilage degradation in the hypomorphic mice was partially induced by the different cartilage volume at the load-bearing regions of the knee joint, we measured total articular cartilage thickness in 6-month-old animals. We found that the thickness of both the medial and lateral tibial plateau was slightly reduced in the hypomorphic mice compared to control; however, the difference was statistically not significant. At the medial plateau, the AC thickness was 128.94 ± 11.33 µm for wild type (*n* = 5 animals), 114.46 ± 15.74 µm for *Agc1^CreERT2/+^* mice (*n* = 7) and 116.96 ± 12.92 µm for *Agc1^CreERT2/CreERT2^* mice (*n* = 6). At the lateral tibial plateau, the AC thickness was 107.18 ± 7.65 µm for wild type, 102.43 ± 5.48 µm for *Agc1^CreERT2/+^* mice and 105.54 ± 10.33 µm for *Agc1^CreERT2/CreERT2^* mice. These data indicate that articular cartilage thickness is apparently not affected by the decreased aggrecan levels, and the thickness of the cartilage does not contribute to cartilage degeneration in the hypomorphic mice *per se*.

### 2.5. Higher Incidence of OA in Agc1^CreERT2/CreERT2^ Mice Is Not Correlated with Increased AC Catabolism

**Figure 5:**
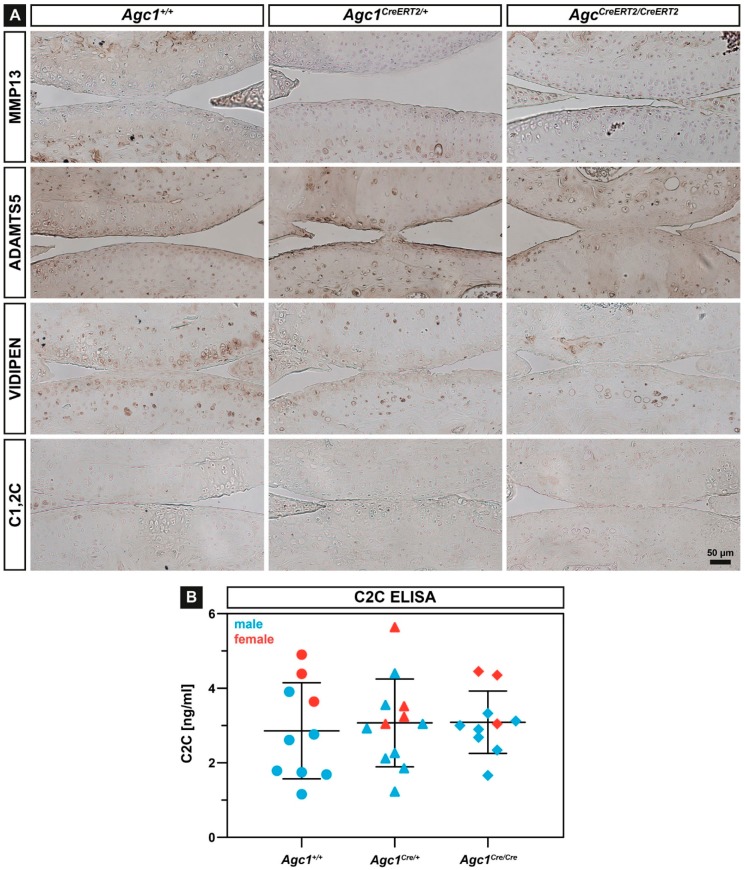
Analysis of cartilage catabolism. (**A**) representative immunostaining of MMP-13, ADAMTS-5, VIDIPEN and C1,2C of sections from the articular cartilage at 12 months of age in *Agc1^+/+^, Agc1^CreERT2/+^* and *Agc1^CreERT2/CreERT2^*, mice. *n* = 5 per genotype; (**B**) C2C ELISA assay in urine of 12-month-old animals. Bars represent mean ± SD. *n* = *Agc1^+/+^*: 6(m)/4(f); *Agc1^Cre/+^*: 8(m)/4(f); *Agc1^Cre/Cre^*: 7(m)/3(f).

The reduced amount of aggrecan and the stiffer biomechanical environment in the articular cartilage might disturb metabolism inducing upregulation of catabolic enzymes which breakdown the ECM. Therefore, we have analyzed the expression of several proteases, which are able to degrade collagens or aggrecan by immunohistochemistry at 12 months of age. We found comparable expression of Matrix Metalloproteinase-13 (MMP-13), a typical collagenase upregulated during OA, and the aggrecanase A Disintegrin and Metalloproteinase with Thrombospondin Motifs-5 (ADAMTS-5) in each genotype (Figure 5A). Similarly, immunostaining for MMP-3, MMP-9 and ADAMTS-4 showed nodifference in the expression of these proteases in the articular cartilage of the *Agc1^CreERT2/CreERT2^*, *Agc1^CreERT2/+^* and *Agc1^+/+^* mice (not shown). Immunostaining with the Collagen Type I and II Cleavage (C1,2C) antibody (Figure 5A) and the Collagen Type II Cleavage (C2C) Elisa assay (Figure 5B), which can detect collagen degradation products on tissue sections and in the urine, respectively, indicated no difference for collagen breakdown between the genotypes. Interestingly, when we stained the articular cartilage sections with an antibody recognizing the MMP-driven aggrecan degradation neo-epitope VIDIPEN, we found gradually decreasing immunoreactivity in *Agc1^CreERT2/+^* and *Agc1^CreERT2/CreERT2^* mice compared to wild type.

## 3. Discussion

The composition and structure of the cartilage extracellular matrix determine the physical properties of the tissue and are pivotal for development of the skeleton and for normal function of the articular cartilage. Disturbance in the assembly, turnover and homeostasis of the ECM proteins can lead to pathological situations resulting in a range of skeletal dysplasias or in degenerative joint diseases such as osteoarthritis. In the cartilage ECM, the collagen fibrillar network entraps proteoglycan aggregates and holds water to resist compressive forces. Aggrecan is the principal proteoglycan component of these aggregates and is essential for the normal function of both transient and permanent cartilaginous tissues. In this study, we have investigated the consequence of decreased aggrecan expression for articular cartilage function by analyzing the hypomorphic *Agc1^CreERT2^* mouse line. We found that reduced aggrecan leads to increased stiffness of the articular cartilage, which in turn results in severe age-associated osteoarthritis.

Mutations in the human aggrecan gene lead to aggrecanopathies, an increasing group of skeletal dysplasias ranging from mild idiopathic short stature to severe chondrodysplasias [9]. Missense mutations typically found in the C-terminal G3 domain of the aggrecan core protein are likely to disrupt interactions with other cartilage ECM proteins. The phenotypic spectrum of these neomorphic mutations includes mild short stature with accelerated bone maturation [12]; osteochondritis dissecans with short stature and early-onset osteoarthritis [11]; and extreme short stature with craniofacial and spinal abnormalities [10]. Heterozygous nonsense mutations accumulated at the G1–G2 and CS1–CS2 domains introduce premature termination codon resulting in the formation of truncated proteins or haploinsufficiency due to nonsense-mediated mRNA degradation [9,31]. These dominant mutations lead to spondyloepiphyseal dysplasia (SED) Kimberly type characterized by proportionate short stature and severe premature osteoarthritis [32] or idiopathic short stature associated with early-onset OA [12,33]. Null or functional null loss of function mutations in mice (*cmd*, *cmd–bc*) [13,14], chicken (nanomelia) [34,35] or cattle (Dexter bulldog dwarfism) [36] are perinatal lethal due to severely abnormal skeletal growth caused by the lack of aggrecan in the cartilage ECM.

Although the findings in human and other species indicate that the absence or reduction of the aggrecan in the cartilage ECM leads to chondrodysplasias and osteoarthritis, the correlation between the level of aggrecan expression and the clinical manifestation of the diseases is not clear. To date, only two mouse models have been investigated in order to link aggrecan gene expression to skeletal phenotype. The naturally occurring cartilage matrix deficiency (*cmd*) is caused by a 7 bp deletion in exon 5 of *Agc1* which leads to a premature stop codon in exon 6 and formation of a short, truncated protein [13]. Homozygous (*cmd/cmd*) mice die at birth due to severe chondrodysplasia characterized by a cleft palate, abnormal tracheal cartilage formation and dwarfism [13] caused by the loss of the normal columnar structure of the growth plate [37]. Heterozygous *cmd/+* mice, however, survive and develop moderate proportional dwarfism at around one month of age. Furthermore, *cmd/+* mice exhibit misalignment of the spine at one year of age, which eventually results in premature death at 19 months due to eating difficulties [15]. In the *Agc1^CreERT2^* mouse model, the 3´ UTR of the *aggrecan* gene was disrupted by knocking in a Cre-ER^T2^ construct to generate an inducible, cartilage-specific deleter mouse line [16]. Although the original publication did not describe a phenotype, it has later been reported that *Agc1^CreERT2/CreERT2^* mice develop dwarfism by one month of age associated with shortening of the growth plate [27]. Now, here we show that the dwarf phenotype exists after the end of skeletal maturation having about 10–20% smaller skeletal elements of the *Agc1^CreERT2/CreERT2^* mice compared to wild type at 6 and 12 months of age (Figure 1). The size reduction affected both the axial and the appendicular skeleton. Interestingly, we observed a mild, but sometimes statistically significant, 3% to 6% reduction of the skeleton and body weight of *Agc1^CreERT2/+^* mice indicating that the skeletally mature heterozygous animals also exhibit a very slight dwarfism.

Insertion of an exogenous targeting construct with selection cassette into intronic or untranslated regions of an endogenous gene could disrupt important regulatory domains, affect splicing or directly influence promoter activity of the endogenous or neighboring genes [38,39,40,41,42]. The interference with the endogenous gene activity leads to hypomorphic alleles with reduced mRNA and protein expression, which is proved to be useful to study the effect of gene doses in various disorders [38,39,43]. It has been previously reported that insertion of the *CreER^T2^* targeting construct into the *Agc1* gene causes the lack of 760 bp of the 3´-UTR. This could affect RNA stability resulting in 20% and 50% reduction of aggrecan mRNA in adult, three-month-old knee epiphyseal cartilage of heterozygous and homozygous knock-in mice, respectively [27]. We found that *Agc1* mRNA levels were reduced in newborn rib cartilage to ~74% in *Agc1^CreERT2/+^* mice and to 64% in *Agc1^CreERT2/CreERT2^* mice compared to the wild type levels (Figure 2A). These observations indicate that disruption of the 3´UTR by the transgene insertion causes a similar reduction of the *Agc1* mRNA levels in chondrocytes regardless of the age or location of the cartilage tissue. In the *cmd* model, the *Agc1* mRNA in embryonic limb cartilage is reduced to 41% in *cmd*/*cmd* mice and to 81% in *cmd/+* mice [15]. Although the direct comparison between the haploinsufficiency *cmd*/*+* model and the hypomorph *Agc1^CreERT2^* model is difficult due to the different nature of the causative mutations, it is reasonable to speculate that about 25–50% reduction in the normal *Agc1* mRNA level is sufficient to generate a mild dwarf phenotype in mice. The reduction of mRNA expression in this range affects the deposition of the aggrecan protein into the ECM as demonstrated by 13% decreased the CS level in spinal cartilage from *cmd*/*+* mice [15], ~50% less aggrecan in knee joint extracts from *Agc1^CreERT2/CreERT2^* mice [27] and the greatly reduced aggrecan immunoreactivity in the articular cartilage of *Agc1^CreERT2/CreERT2^* knee joints (Figure 2B).

The most dramatic phenotype we found in the adult *Agc1^CreERT2/CreERT2^* mice is the severe degeneration of the knee articular cartilage typical for late stage osteoarthritis (Figure 4). Since OA was not described in aging *cdm*/*+* mice, we hypothetise that *Agc1^CreERT2/CreERT2^* mice have a greater loss of aggrecan in the articular cartilage which impairs its function and eventually leads to the complete erosion of the AC surface in males by 12 months of age. The aggrecan–hyaluronan aggregates have a crucial role for maintaining the biomechanical properties of the articular cartilage by immobilizing a vast amount of negative charges and generating an osmotic swelling pressure, which provides the tissue with resistance against compressive forces [3]. *Agc1^CreERT2/CreERT2^* mice displayed reduced aggrecan and GAG content in the knee joint suggesting alterations in the biomechanics of the articular cartilage. Applying nanoscale IT-AFM, we observed prominent stiffening of the AC from *Agc1^CreERT2/CreERT2^* mice (Figure 3B) at six months of age, when histological differences of AC degeneration were not evident between the genotypes (Figure 4B). The increased stiffness was detected in the superficial, middle and deep zones implying a systemic hardening of the whole articular cartilage.

In normal cartilage, the swelling pressure is balanced by the tensile force of the collagen network and modifying the collagen fibril-hydrated proteoglycan ratio could affect the swelling and biomechanical properties of the tissue. Earlier studies have shown that porcine articular cartilage incubated in hypotonic salt solution or digested with proteoglycan-degrading enzymes has increased cartilage nanostiffness [28,44]. Accordingly, the reduced negative charge density of the sGAG chains due to the decreased aggrecan level in the homozygous hypomorphic mice could result in reduced tissue hydration, decreased swelling pressure and increased constraining matrix stress, which in turn elevates cartilage stiffness. It is important to note, however, that the imbalance between osmotic swelling pressure and the tensile forces apparently does not affect cartilage volume, as the thickness of the tibial articular cartilage was not significantly altered in the *Agc1^CreERT2/CreERT2^* mice.

We have previously shown that nanoscale indentation is a sensitive tool to assess cartilage biomechanical properties of the growth plate, the articular cartilage and the intervertebral disc [29,30,45,46,47]. We showed that IT-AFM depicts subtle biomechanical differences between wild type and genetically modified mice carrying mutations, which affect the collagen network [45,46,47] or the proteoglycans [30]. The method is capable of resolving the biomechanical properties of the collagen fibrils and the entrapped proteoglycan gel resulting in a bimodal stiffness distribution on the recorded histograms [28,29]. We observed a shift for the stiffness of both the collagens and the proteoglycans in the *Agc1^CreERT2/CreERT2^* mice compared to wild type and *Agc1^CreERT2/+^* mice. This general increase in stiffness of the major components of the cartilage matrix could lead to a rigid AC surface, which is more susceptible for mechanical load-induced degradation. We have previously found that mice lacking collagen IX, an essential component of the heterotypic cartilage collagen fibrils, also display increased nanostiffness of the hip articular cartilage before obvious signs of joint degeneration and the onset of osteoarthritis [45]. In human AC, nanostiffness is increasing by age due to GAG loss-induced dehydration of the proteoglycan moiety [45] or stiffening of the collagen fibrils [48] and could predispose for OA. However, disintegration of the cartilage extracellular matrix (e.g., the collagen meshwork) during the onset and progression of OA is accompanied by decreased compressive stiffness in both animal models and human patients [45,49,50].

During the course of OA, usually degradation of aggrecan followed by breakdown of the collagen network are critical steps for progressive cartilage destruction. In *Agc1^CreERT2^/^CreERT2^* mice, the reduced level of aggrecan apparently does not affect cartilage metabolism as demonstrated by the normal expression of MMP-13, ADAMTS-5 and collagen and aggrecan degradation neoepitopes (Figure 5). These findings suggest a physical activity-induced “wear and tear” mechanism for destruction of the mechanically abnormal AC in the *Agc1^CreERT2^/^CreERT2^* mice.

Epidemiological studies have shown that the prevalence, incidence and severity of osteoarthritis are higher in postmenopausal women than men due to hormonal, anatomical and genetic factors [51,52]. In murine, however, sex hormones, body weight, behavior and activity differences between genders lead to higher incidence of joint degeneration in male mice in both age-associated and experimentally-induced osteoarthritis models [53,54,55]. In line with this evidence, the cartilage destruction was more severe in male *Agc1^CreERT2/CreERT2^* mice than in female mice implying that physical activities (e.g., fighting) and high body weight could worsen the condition.

In summary, we have demonstrated that the hypomorphic *Agc1^CreERT2/CreERT2^* mice develop age-associated OA characterized by severe erosion of the articular cartilage by 12 months of age. The phenotype was associated with stiffening of the AC, indicating that aggrecan level crucially affects biomechanical properties of the cartilage matrix. As heterozygous mice occasionally show a tendency for reduced length of skeletal elements and increased cartilage destruction, we advise the cartilage field for a careful interpretation of experiments with cartilage specific-deletion of floxed genes using the *Agc1^CreERT2^* line. On the other hand, these hypomorphic mice are ideal models to study aggrecan gene doses and skeletal phenotype correlation through the entire postnatal life with implications for human aggrecanopathies.

## 4. Materials and Methods

### 4.1. Mouse Model and Husbandry

*Agc1^tm(IRES-CreERT2)^* [16] were purchased from the Jackson Laboratory (JAX stock #019148). Upon arrival, mice were bred with 129/Sv outbred mice for at least two generations. Mice were housed in groups of 2–4 per cage and received food and water *ad libitum*. They were maintained in a temperature- and air- controlled environment, with 12 h light/dark cycle. Animals were handled and housed according to the federal and institutional guidelines for the care and use of laboratory animals, approved by the Central Animal Facility of the LMU Munich and the government of Upper Bavaria (Application number: 55.2-1-54-2532-15-2016). For the sake of simplicity, control, heterozygous and homozygous mice are denoted as *Agc1^+/+^*, *Agc1^CreERT2/+^* and *Agc1^CreERT2/CreERT2^*, respectively. Newborn, 6- and 12-month-old male and female mice were included in the study. Genotyping was performed according to the Jackson Laboratory protocol. Mice were sacrificed via cervical dislocation and tissue collection performed *post mortem*.

### 4.2. Radiography and Morphometry

X-ray images of euthanized mice were taken with a Faxitron X-ray cabinet (model 43855A) at 35 kV, 2 mA and 2 sec exposure time. Total body length (from the snout to the end of the tail) and the length of the skeletal elements (4^th^ lumbar vertebra, tibia, femur and humerus) were analyzed with the distance measurement plug-in of the syngo Imaging XS-VA60B software (Siemens, Erlangen, Germany).

### 4.3. Sample Preparation

Hind limbs of euthanized mice were de-skinned and partially cleaned from the muscles around the knee joints. For histology and immunohistochemistry, knee joints from the left side were fixed in 4% paraformaldehyde (PFA) in phosphate buffered saline (PBS) pH 7.4 (Sigma-Aldrich, Taufkirchen, Germany), overnight at 4 °C. The next day, samples were washed 3 times in PBS and afterwards decalcified for approximately 4 weeks in a 20% ethylenediaminetetraacetic acid (EDTA)/PBS pH 8.0 solution (Sigma-Aldrich). Decalcified specimens were washed 3 times in PBS, then dehydrated through an ascending row of ethanol, cleared in xylol and finally embedded in paraffin. Samples were cut on a Rotary Microtome HM360 in 8 µm sagittal sections and placed on Superfrost plus glass slides (Thermo Scientific, Madison, WI, USA). For AFM measurements, the correspondent right knee joints were directly enclosed in Tissue-Tek cryomedia (Sakura, Zoeterwoude, NL, USA) on the base of plastic disposable cassettes, and gradually frozen by transferring them on a chilled copper plate placed on dry ice. Sagittal sections of 30 µm were cut with the aid of adhesive tape (as described in [29]) using a Microm HM500 cyostat (Thermo Scientific) and kept at −20 °C until use.

### 4.4. Histology

For the histological examination of proteoglycan content, Toluidine blue and Safranin O/Fast Green staining were performed. For Toluidine blue staining, tissue sections were deparaffinized, rehydrated and incubated for 10 min in 0.1% Toluidine blue in toluidine buffer pH 2.5. Excess stain, was adsorbed with Whatman paper and then slides were immersed in a 2% K_3_Fe(CN_6_)/dH2O solution for 3 min. Finally, sections were directly mount with Roti-histokit (Roth, Karlsruhe, Germany). For Safranin O/Fast Green staining a 0.5% hematoxylin staining solution (Roth) was used for 2 min and then washed with tap water for 5 min. After dipping in 1% acetic alcohol (Merck, Darmstadt, Germany). slides were incubated in a 0.02% fast green solution (Sigma-Aldrich) for 1 min. Next, tissue sections were immersed in 1% acetic acid for 30 s and then a 1% Safranin O solution (Sigma-Aldrich) was applied for 30 min. After three changes of 95% ethanol, and two of 100% ethanol, sections were cleared in xylol and mounted with Roti-Histokit. The thickness of the tibial articular cartilage in 6-month-old animals was measured as previously described [56]. Briefly, representative micrographs of Safranin O/Fast Green sections from the lateral and medial tibial plateau were analyzed by using the linear measure tool of the ZEN 2.3 Lite image capturing and processing software (Carl Zeiss, Jena, Germany). In each section, three regions of the articular cartilage extending from the surface to the subchondral bone were measured. The mean of the three values was used to characterize the total thickness of the articular cartilage. At least five animals of each genotype were evaluated.

### 4.5. Scoring of Articular Cartilage Erosion

The pathological scoring of AC has been performed on Safranin O/Fast Green stained sagittal tissue sections. To have a full representation of the entire knee, about 10 slides, harvested at approximately 80 µm intervals, were chosen. Slides were evaluated by two independent observers in a blinded fashion. To assess cartilage degeneration, the OARSI scoring system was used [57]. Evaluation of articular cartilage structure comprehends eight grades as follows: 0—normal cartilage; 0.5—loss of proteoglycan stain without cartilage damage; 1—Surface fibrillations; 2—Cleft extending just below the superficial zone; 3—Cleft/erosion to the calcified cartilage extending to < 25% of the articular surface; 4—25–50% loss of articular surface; 5—50–75% loss of articular surface; 6—Eburnation with > 75% loss of cartilage. The means of the two independent observers of the maximal histopathological scores obtained across the entire knee joints were presented 

### 4.6. Immunohistochemistry

For immunohistochemistry, paraffin embedded sections were deparaffinized, rehydrated and digested with 2 mg/mL bovine testicular hyaluronidase/PBS pH 5.0 at 37 °C for 30 min. Endogenous peroxidase activity was quenched with a 0.3% H_2_O_2_/methanol solution for 30 min. Sections were blocked with 1% bovine serum albumin/PBS for 60 min at room temperature. Afterwards, slides were incubated overnight at 4 °C with the following antibodies diluted in blocking solution: aggrecan (Millipore ab1031, 1:200, Beverly, MA, USA), collagen type II (DSHB CIICI, 5 µg/mL), MMP-13 (Sigma Aldrich MAB13424, 1:100), ADAMTS-5 (Abcam ab13976, 1:500, Cambridge, MA, USA), VIDIPEN (a gift of Amanda Fosang, University of Melbourne, 1:1000), and C1,2C (IBEX 50-1035, 1:400). The next day, sections were incubated with the correspondent biotinylated secondary antibodies and immunostained by the avidin-biotin complex (Vectastain ABC Elite kit) method or the Vector M.O.M kit (both Vector Laboratories, Burlingame, CA, USA) and the 3,3’-diaminobenzidine (Sigma) as chromogenic substrate. Finally, sections were counterstained with hematoxylin and mounted with Roti-Histokit.

### 4.7. RNA Isolation and Quantitative RT-PCR

Total RNA was isolated from primary chondrocytes of newborn mice. Briefly, rib cages from euthanized mice were dissected, cleaned from the attached tissues with forceps and digested for 30 min in a solution of 2 mg/mL collagenase type 2 (Worthington, Malvern, PA, USA) in Dulbecco’s Modified Eagle’s (DMEM)/Ham’s F12 1:1 (Thermo Fischer Scientific) supplemented with 1× penicillin/streptomycin (Biochrom, Berlin, Germany). After the first enzymatic digestion, the perichondrium was removed and cleaned ribs were re-placed in a new collagenase solution for 3 h at 37 °C in a humidified incubator with 5% CO_2_. The obtained cell suspension was centrifuged at 500 g for 5 min and washed with PBS. Total RNA isolation and cDNA synthesis were performed with the Qiagen RNeasy Mini kit (Qiagen, Hilden, Germany) and the Transcriptor First-Strand cDNA Synthesis kit (Roche, Basel, Switzerland), respectively, according to the manufacturer’s instructions. Quantitative RT-PCR was performed using the gene specific TaqMan probes from Integrated DNA Technologies for *glyceraldehyde 3-phosphate dehydrogenase (GAPDH)* (Mm.PT.39a.1) and *aggrecan* (Mm.PT.58.10174685). PrimeTime Gene Expression Master Mix, dH2O and cDNA samples were pipetted in triplicates in a 96-well plate and a PCR reaction was carried out in a LightCycler 96 thermocycler provided with the LightCycler 96 Software (Roche). The relative gene expression is calculated against the housekeeping gene *GAPDH* by the comparative ΔCt method. Results in graph show the mean ± standard deviation of two PCR runs from three mice per genotypes.

### 4.8. Atomic Force Microscopy

Nano-scale indentation-type atomic force microscopy (IT-AFM) was carried out using a JPK NanoWizard® 1 (JPK Instruments, Berlin, Germany) mounted on an inverse Axiovert 200 optical microscope (Carl Zeiss, Jena, Germany) to enable a precise positioning of the AFM tip on the region of interest. For measurements, silicon nitride cantilevers (MLCT, Cantilever E, Bruker) with a nominal spring constant of 0.1 N/m and pyramidal tips with a nominal radius of 20 nm were used. All IT-AFM measurements were carried out in PBS at pH 7.4. In order to determine the actual cantilever spring constant, each cantilever was individually calibrated using the thermal noise method [58]. Every spring constant determination was repeated three times and the mean value was used for data analysis. In each cartilage zone (superficial, middle and deep) an area of 3 × 3 µm, 25 × 25 force-indentation curves were recorded with a constant vertical tip velocity of 15 µm/s and a sampling rate of 5 kHz. For data analysis, the JPK Data Processing Software was used (Version 6.1.102, JPK Instruments). The Young’s modulus was extracted from the approach section of the force-indentation curves from the contact point up to a maximum indentation depth of 500 nm, using a modified Hertz model for a pyramidal indenter as described in [29,59]. For comparison, stiffness distributions (histograms) were calculated for each developmental stage and genotype. Two sections per animal were scanned with a total of 3 × 625 force-indentation curves for each cartilage zone. To locate the maxima of these histograms, a linear combination of two Gaussian distributions was fitted to each histogram using the Igor Pro software (Version 6.3.7.2, WaveMetrics, Portland, OR, USA).

### 4.9. Enzyme-Linked Immunosorbent Assay

In order to detect the levels of collagen type 2 degradation products, the commercially available Mouse Collagen Type 2 Cleavage ELISA kit (MyBioSource, MBS725817, San Diego, CA, USA) was used according to the manufacturer instructions. Urine samples were collected from 12-month-old mice on the day of sacrification and kept at −80 °C until use. C2C levels are expressed in ng/mL and were normalized to urinary creatinine concentration. Quantitative determination of creatinine in urine samples was assessed with the Mouse Creatinine kit from Crystal Chem (Cat. 80350) as per manufacturer protocol.

### 4.10. Statistical Analysis

Statistical significance was calculated after determination of the Gaussian distribution using a one-way ANOVA test (GraphPad Prism, San Diego, CA, USA) with appropriate post hoc tests. Statistical significance was assumed at a *p*-value of ≤ 0.05. All values represent the mean and the standard deviation (SD). In all experiments, at least three animals were used (a list of the precise number of animals used in each experiment is stated in figure legends).

## 5. Conclusions

In conclusion, our data demonstrate that *Agc1^CreERT2/CreERT2^* mice have smaller skeleton, stiffer articular cartilage and are prone to spontaneous, age-associated osteoarthritis owing to reduced deposition of aggrecan in cartilage. Therefore, the homozygous *Agc1^CreERT2/CreERT2^* mice should not be used for conditional gene ablation experiments assessing gene function in the articular cartilage. However, the *Agc1^CreERT2/CreERT2^* mice could serve as valuable model for studying aggrecanopathies caused by reduced aggrecan expression in the cartilaginous skeleton.
